# Dr. Uri Colvin Lynde

**Published:** 1916-12

**Authors:** 


**Dr. Uri Colvin Lynde,** Buffalo 1859, died at his home in Buffalo, Nov. 21, aged 83. Death is ascribed to the remote effects of a fracture of the skull sustained in 1910 by falling from a trolley. He was born in Concord, Erie Co., N. Y., and was graduated from the Buffalo Central High School. He served as assistant surgeon to the 116th N. Y. Regiment of Volunteers for 18 months, 1862-3, when he was given sick leave. After the War, he took a post-graduate course in surgery at Jefferson Medical College, Philadelphia. From that time on, he was a prominent practitioner of Buffalo and was, at the time of his death, one of its oldest members. He was a classmate of the late Lt. Col. William Warren Potter, editor of this journal. Only two members of this class are now alive.

